# Supplementary material for: Modeling glioblastoma heterogeneity as a dynamic network of cell states
Source: Mol Syst Biol. 2021 Sep 16;17(9):e10105. doi: 10.15252/msb.202010105 (PMC8444284; doi:10.15252/msb.202010105)
Supplement: Supplementary file 6 — Source Data for Figure 5 [file MSB-17-e10105-s004.zip › Figure5A_sourcedata/GSEA_3017/nosigdb_stateE.GseaPreranked.1622795031093/RICHARDS_NOWAKOWSKI_SCIENCE_2017_ENDOTHELIAL_UPREG.html]

Details for gene set RICHARDS\_NOWAKOWSKI\_SCIENCE\_2017\_ENDOTHELIAL\_UPREG[GSEA]

|  || Dataset | state63017 |
| Phenotype | NoPhenotypeAvailable |
| Upregulated in class | na\_pos |
| GeneSet | RICHARDS\_NOWAKOWSKI\_SCIENCE\_2017\_ENDOTHELIAL\_UPREG |
| Enrichment Score (ES) | 0.46326402 |
| Normalized Enrichment Score (NES) | 3.7112775 |
| Nominal p-value | 0.0 |
| FDR q-value | 0.0 |
| FWER p-Value | 0.0 |
Table: GSEA Results Summary

  

Fig 1: Enrichment plot: RICHARDS\_NOWAKOWSKI\_SCIENCE\_2017\_ENDOTHELIAL\_UPREG      
 Profile of the Running ES Score & Positions of GeneSet Members on the Rank Ordered List

  

| PROBE | GENE SYMBOL | GENE\_TITLE | RANK IN GENE LIST | RANK METRIC SCORE | RUNNING ES | CORE ENRICHMENT || 1 | PMP22 |  |  | 0 | 1.393 | 0.0266 | Yes |
| 2 | CTGF |  |  | 2 | 1.276 | 0.0498 | Yes |
| 3 | CYR61 |  |  | 3 | 1.169 | 0.0721 | Yes |
| 4 | CCL2 |  |  | 5 | 0.975 | 0.0895 | Yes |
| 5 | ID3 |  |  | 6 | 0.972 | 0.1080 | Yes |
| 6 | SPARCL1 |  |  | 7 | 0.963 | 0.1264 | Yes |
| 7 | PODXL |  |  | 8 | 0.959 | 0.1448 | Yes |
| 8 | ANXA1 |  |  | 12 | 0.826 | 0.1569 | Yes |
| 9 | CEBPD |  |  | 15 | 0.791 | 0.1696 | Yes |
| 10 | RCAN1 |  |  | 17 | 0.750 | 0.1827 | Yes |
| 11 | ANXA2 |  |  | 18 | 0.733 | 0.1967 | Yes |
| 12 | PRSS23 |  |  | 21 | 0.722 | 0.2081 | Yes |
| 13 | CNN3 |  |  | 24 | 0.702 | 0.2190 | Yes |
| 14 | IFITM3 |  |  | 26 | 0.682 | 0.2309 | Yes |
| 15 | TAGLN2 |  |  | 32 | 0.621 | 0.2367 | Yes |
| 16 | GJA1 |  |  | 33 | 0.618 | 0.2485 | Yes |
| 17 | NEDD9 |  |  | 38 | 0.590 | 0.2549 | Yes |
| 18 | EMP1 |  |  | 40 | 0.584 | 0.2649 | Yes |
| 19 | CALD1 |  |  | 41 | 0.580 | 0.2759 | Yes |
| 20 | PMEPA1 |  |  | 44 | 0.544 | 0.2839 | Yes |
| 21 | ID1 |  |  | 45 | 0.542 | 0.2943 | Yes |
| 22 | IFITM2 |  |  | 46 | 0.536 | 0.3045 | Yes |
| 23 | FRMD4B |  |  | 48 | 0.532 | 0.3134 | Yes |
| 24 | S100A10 |  |  | 51 | 0.527 | 0.3211 | Yes |
| 25 | SERPINE1 |  |  | 52 | 0.526 | 0.3311 | Yes |
| 26 | WLS |  |  | 65 | 0.472 | 0.3256 | Yes |
| 27 | CDH6 |  |  | 70 | 0.453 | 0.3294 | Yes |
| 28 | SMAD1 |  |  | 72 | 0.450 | 0.3368 | Yes |
| 29 | HSPA5 |  |  | 73 | 0.449 | 0.3454 | Yes |
| 30 | SERPINB6 |  |  | 77 | 0.440 | 0.3502 | Yes |
| 31 | UPP1 |  |  | 78 | 0.438 | 0.3585 | Yes |
| 32 | CST3 |  |  | 86 | 0.425 | 0.3582 | Yes |
| 33 | CD59 |  |  | 89 | 0.418 | 0.3638 | Yes |
| 34 | TMSB4X |  |  | 93 | 0.412 | 0.3680 | Yes |
| 35 | ATF3 |  |  | 101 | 0.397 | 0.3671 | Yes |
| 36 | TIMP3 |  |  | 106 | 0.391 | 0.3697 | Yes |
| 37 | CD99 |  |  | 107 | 0.389 | 0.3772 | Yes |
| 38 | LGALS1 |  |  | 113 | 0.368 | 0.3781 | Yes |
| 39 | FNDC3B |  |  | 123 | 0.354 | 0.3740 | Yes |
| 40 | AHNAK |  |  | 125 | 0.351 | 0.3795 | Yes |
| 41 | VMP1 |  |  | 127 | 0.351 | 0.3850 | Yes |
| 42 | DSTN |  |  | 129 | 0.348 | 0.3904 | Yes |
| 43 | SEMA6A |  |  | 130 | 0.348 | 0.3971 | Yes |
| 44 | PDGFB |  |  | 131 | 0.347 | 0.4037 | Yes |
| 45 | SOCS3 |  |  | 141 | 0.342 | 0.3994 | Yes |
| 46 | RHOB |  |  | 148 | 0.335 | 0.3985 | Yes |
| 47 | PDLIM2 |  |  | 155 | 0.329 | 0.3976 | Yes |
| 48 | PRDM1 |  |  | 156 | 0.327 | 0.4038 | Yes |
| 49 | MACF1 |  |  | 160 | 0.326 | 0.4064 | Yes |
| 50 | ZFP36L2 |  |  | 162 | 0.324 | 0.4114 | Yes |
| 51 | SPATS2L |  |  | 163 | 0.323 | 0.4175 | Yes |
| 52 | FILIP1L |  |  | 167 | 0.320 | 0.4200 | Yes |
| 53 | JUN |  |  | 172 | 0.316 | 0.4212 | Yes |
| 54 | MBNL2 |  |  | 173 | 0.316 | 0.4273 | Yes |
| 55 | ATXN1 |  |  | 174 | 0.315 | 0.4333 | Yes |
| 56 | ARHGAP29 |  |  | 175 | 0.314 | 0.4393 | Yes |
| 57 | A2M |  |  | 184 | 0.309 | 0.4355 | Yes |
| 58 | SPTBN1 |  |  | 194 | 0.300 | 0.4303 | Yes |
| 59 | SDC3 |  |  | 200 | 0.295 | 0.4299 | Yes |
| 60 | CAST |  |  | 202 | 0.294 | 0.4343 | Yes |
| 61 | SAT1 |  |  | 206 | 0.291 | 0.4363 | Yes |
| 62 | ACSL3 |  |  | 207 | 0.291 | 0.4418 | Yes |
| 63 | GNG11 |  |  | 210 | 0.289 | 0.4449 | Yes |
| 64 | PLEKHG1 |  |  | 217 | 0.284 | 0.4431 | Yes |
| 65 | FERMT2 |  |  | 219 | 0.283 | 0.4473 | Yes |
| 66 | PTTG1IP |  |  | 225 | 0.280 | 0.4466 | Yes |
| 67 | GUK1 |  |  | 231 | 0.277 | 0.4458 | Yes |
| 68 | CPM |  |  | 236 | 0.271 | 0.4462 | Yes |
| 69 | EHD2 |  |  | 240 | 0.267 | 0.4476 | Yes |
| 70 | QKI |  |  | 241 | 0.267 | 0.4527 | Yes |
| 71 | CHMP2A |  |  | 243 | 0.266 | 0.4566 | Yes |
| 72 | TRIM56 |  |  | 248 | 0.263 | 0.4568 | Yes |
| 73 | INSIG1 |  |  | 252 | 0.261 | 0.4581 | Yes |
| 74 | TRAM2 |  |  | 256 | 0.259 | 0.4595 | Yes |
| 75 | LEPROT |  |  | 259 | 0.259 | 0.4620 | Yes |
| 76 | WWTR1 |  |  | 263 | 0.256 | 0.4633 | Yes |
| 77 | EVA1C |  |  | 270 | 0.250 | 0.4608 | No |
| 78 | DONSON |  |  | 274 | -0.251 | 0.4619 | No |
| 79 | ZFP36L1 |  |  | 287 | -0.255 | 0.4523 | No |
| 80 | NSUN6 |  |  | 288 | -0.256 | 0.4572 | No |
| 81 | CNTLN |  |  | 326 | -0.264 | 0.4175 | No |
| 82 | HSPD1 |  |  | 329 | -0.264 | 0.4201 | No |
| 83 | SLK |  |  | 343 | -0.266 | 0.4095 | No |
| 84 | APOLD1 |  |  | 374 | -0.275 | 0.3785 | No |
| 85 | SLC12A2 |  |  | 389 | -0.279 | 0.3669 | No |
| 86 | HSPA8 |  |  | 392 | -0.280 | 0.3698 | No |
| 87 | GDI2 |  |  | 401 | -0.283 | 0.3655 | No |
| 88 | DERA |  |  | 402 | -0.284 | 0.3709 | No |
| 89 | TXNIP |  |  | 404 | -0.284 | 0.3751 | No |
| 90 | CHCHD1 |  |  | 417 | -0.289 | 0.3661 | No |
| 91 | CDK2 |  |  | 422 | -0.290 | 0.3669 | No |
| 92 | TPP2 |  |  | 436 | -0.295 | 0.3568 | No |
| 93 | LSM8 |  |  | 441 | -0.297 | 0.3576 | No |
| 94 | PNRC2 |  |  | 461 | -0.304 | 0.3404 | No |
| 95 | UPF2 |  |  | 465 | -0.304 | 0.3426 | No |
| 96 | EPB41L2 |  |  | 488 | -0.310 | 0.3219 | No |
| 97 | PRDX3 |  |  | 489 | -0.310 | 0.3278 | No |
| 98 | KLHL4 |  |  | 496 | -0.313 | 0.3265 | No |
| 99 | COX17 |  |  | 505 | -0.316 | 0.3229 | No |
| 100 | RBPMS |  |  | 519 | -0.320 | 0.3133 | No |
| 101 | FKBP5 |  |  | 536 | -0.328 | 0.3002 | No |
| 102 | RBM17 |  |  | 546 | -0.331 | 0.2956 | No |
| 103 | MCM3 |  |  | 560 | -0.340 | 0.2864 | No |
| 104 | CKLF |  |  | 574 | -0.347 | 0.2773 | No |
| 105 | JMJD1C |  |  | 597 | -0.356 | 0.2575 | No |
| 106 | SNHG5 |  |  | 605 | -0.360 | 0.2559 | No |
| 107 | ZEB1 |  |  | 653 | -0.396 | 0.2066 | No |
| 108 | EGR1 |  |  | 656 | -0.398 | 0.2118 | No |
| 109 | SAE1 |  |  | 668 | -0.408 | 0.2063 | No |
| 110 | SLC25A5 |  |  | 695 | -0.426 | 0.1830 | No |
| 111 | MAP3K1 |  |  | 702 | -0.431 | 0.1840 | No |
| 112 | SLC20A1 |  |  | 717 | -0.444 | 0.1755 | No |
| 113 | GSTO1 |  |  | 731 | -0.459 | 0.1686 | No |
| 114 | DCP2 |  |  | 762 | -0.500 | 0.1419 | No |
| 115 | KIF5B |  |  | 770 | -0.515 | 0.1432 | No |
| 116 | CDC45 |  |  | 773 | -0.521 | 0.1508 | No |
| 117 | CREM |  |  | 820 | -0.611 | 0.1068 | No |
| 118 | TM4SF1 |  |  | 823 | -0.619 | 0.1162 | No |
| 119 | FAM111A |  |  | 858 | -0.727 | 0.0890 | No |
| 120 | H2AFZ |  |  | 893 | -0.849 | 0.0641 | No |
Table: GSEA details [plain text format]

  

Fig 2: RICHARDS\_NOWAKOWSKI\_SCIENCE\_2017\_ENDOTHELIAL\_UPREG: Random ES distribution      
 Gene set null distribution of ES for **RICHARDS\_NOWAKOWSKI\_SCIENCE\_2017\_ENDOTHELIAL\_UPREG**
